# Uridine Diphosphate-Dependent Glycosyltransferases from *Bacillus subtilis* ATCC 6633 Catalyze the 15-*O*-Glycosylation of Ganoderic Acid A

**DOI:** 10.3390/ijms19113469

**Published:** 2018-11-05

**Authors:** Te-Sheng Chang, Jiumn-Yih Wu, Tzi-Yuan Wang, Kun-Yuan Wu, Chien-Min Chiang

**Affiliations:** 1 Department of Biological Sciences and Technology, National University of Tainan, Tainan 70005, Taiwan; mozyme2001@gmail.com (T.-S.C.); joy0067tw@gmail.com (K.-Y.W.); 2 Department of Food Science, National Quemoy University, Kinmen County 892, Taiwan; wujy@nqu.edu.tw; 3 Biodiversity Research Center, Academia Sinica, Taipei 115, Taiwan; tziyuan@gmail.com; 4 Department of Biotechnology, Chia Nan University of Pharmacy and Science, No. 60, Sec. 1, Erh-Jen Rd., Jen-Te District, Tainan 71710, Taiwan

**Keywords:** ganoderic acid, *Bacillus subtilis*, biotransformation, UDP-glycosyltransferase

## Abstract

*Bacillus subtilis* ATCC (American type culture collection) 6633 was found to biotransform ganoderic acid A (GAA), which is a major lanostane triterpenoid from the medicinal fungus *Ganoderma lucidum*. Five glycosyltransferase family 1 (GT1) genes of this bacterium, including two uridine diphosphate-dependent glycosyltransferase (UGT) genes, *BsUGT398* and *BsUGT489*, were cloned and overexpressed in *Escherichia coli*. Ultra-performance liquid chromatography confirmed the two purified UGT proteins biotransform ganoderic acid A into a metabolite, while the other three purified GT1 proteins cannot biotransform GAA. The optimal enzyme activities of BsUGT398 and BsUGT489 were at pH 8.0 with 10 mM of magnesium or calcium ion. In addition, no candidates showed biotransformation activity toward antcin K, which is a major ergostane triterpenoid from the fruiting bodies of *Antrodia cinnamomea*. One biotransformed metabolite from each BsUGT enzyme was then isolated with preparative high-performance liquid chromatography. The isolated metabolite from each BsUGT was identified as ganoderic acid A-15-*O*-β-glucoside by mass and nuclear magnetic resonance spectroscopy. The two BsUGTs in the present study are the first identified enzymes that catalyze the 15-*O*-glycosylation of triterpenoids.

## 1. Introduction

The fundamental step in developing new drugs is finding new compounds. In addition to synthetic and natural sources, biotransformation is an alternative route for obtaining new compounds. Xenobiotics can be biotransformed into new compounds via microorganisms or enzymes. Based on the numerous bioactivities of triterpenoids, several scientists have focused on studying triterpenoid biotransformations to find new bioactive triterpenoids [1,2,3,4]. 

*Ganoderma lucidum* (the Chinese name for which is “Lingzhi”) has numerous bioactive constituents, such as polysaccharides and triterpenoids [5]; more than 300 different triterpenoids have been identified from *Ganoderma* spp. [6]. Ganoderic acid A (GAA) is a typical *Ganoderma* triterpenoid [7] which has been validated with many bioactivities [8,9,10,11,12,13]. Although hundreds of bioactive triterpenoids have been isolated from *G. lucidum*, few studies have focused on the biotransformation of *Ganoderma* triterpenoids. On the other hand, our previous study showed that *Bacillus subtilis* ATCC (American type culture collection) 6633 biotransforms antcin K, a major ergostane triterpenoid from the fruiting bodies of *Antrodia cinnamomea*, to its glycoside derivatives [14]. In the present study, the *B. subtilis* strain was confirmed to biotransform the most abundant *Ganoderma* triterpenoid GAA as well. Therefore, we were interested in examining the key enzymes of *B. subtilis* ATCC 6633 in the biotransformation of GAA and antcin K triterpenoids.

In nature, glycosylation is catalyzed by glycosyltransferases (GTs, EC 2.4.x.y), which transfer sugar moieties from the activated donor molecules to specific acceptor molecules [15,16,17]. According to the Carbohydrate-Active Enzymes (CAZy) database, GTs are now classified into 105 families [18]. Among them, some glycosyltransferases family 1 (GT1) proteins were found to use small molecules, such as flavonoids or terpenes, as sugar acceptors [15,16,17]. Furthermore, uridine diphosphate (UDP)-dependent glycosyltransferases (UGTs), which belong to the GT1 gene family, can use UDP-glucose as the sugar donor [15,18]. Thus, five GT1 genes, including two UGT genes, were cloned from the *B. subtilis* ATCC 6633 strain and overexpressed in *Escherichia coli*. The biotransformation activities of the expressed enzymes were confirmed and optimized in this study. The metabolite biotransformed by the functional enzymes was then isolated and identified.

## 2. Results

### 2.1. Confirming Biotransformation of GAA by Bacillus Subtilis ATCC 6633

To confirm whether *B. subtilis* ATCC 6633 could biotransform GAA, the bacterium was cultivated in broth with GAA, and the fermentation broth was analyzed using ultra-performance liquid chromatography (UPLC).

**Figure 1:**
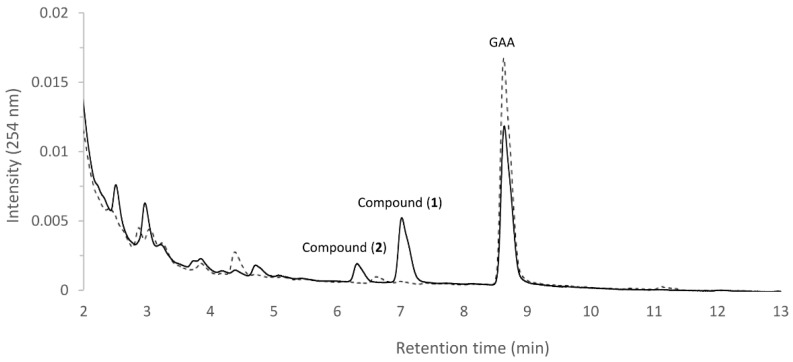
Biotransformation of ganoderic acid A (GAA) by *B. subtilis* ATCC (American type culture collection) 6633. The strain was cultivated in modified glucose-nutrient (MGN) media containing 0.02 mg/mL of ganoderic acid A (GAA). The initial cultivation (dashed line) and the 72 h cultivation (solid line) of the fermentation broth were analyzed with ultra-performance liquid chromatography (UPLC). The UPLC operation conditions are described in the Materials and Methods section.

Figure 1 shows the UPLC of the initial (dashed line) and 24 h (solid line) fermentation broths of the strain *B. subtilis* ATCC 6633 with GAA. The precursor GAA appeared at a retention time (RT) of 8.6 min. After 24 h fermentation, the peak of the precursor decreased, while two new peaks—a major peak, compound (**1**), with a RT of 7.0 min and a minor peak, compound (**2**) with a RT of 6.3 min—appeared. Moreover, the two new peaks did not appear at 24 h of the fermentation broths of the strain in the absence of GAA (Figure S1). The results confirmed that GAA was biotransformed by the strain *B. subtilis* ATCC 6633.

### 2.2. Phylogenetic Analysis of GTs from B. subtilis ATCC 6633

**Figure 2:**
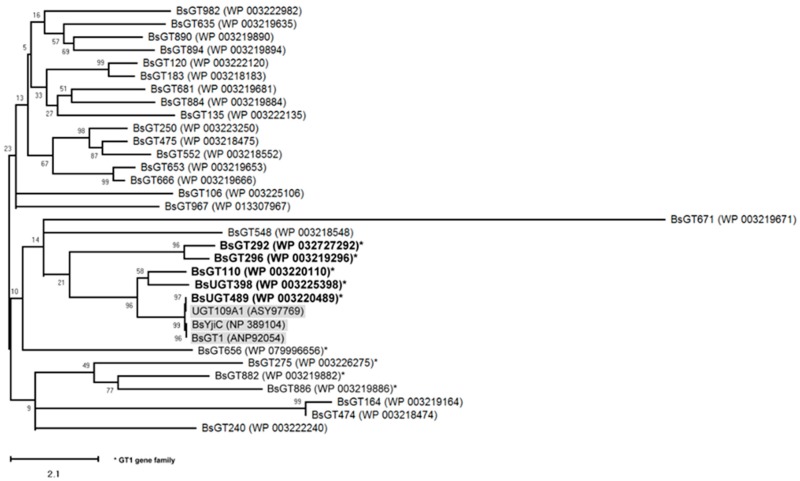
Molecular phylogenetic analysis of BsGT candidates inferred with the maximum likelihood (ML) method. The best-fit ML model selection is mtREV24 [23]. The tree with the highest log likelihood (−6498.11) is shown. The percentage of trees in which the associated taxa clustered together is shown next to the branches. Initial tree(s) for the heuristic search were obtained automatically by applying the neighbor-join and BioNJ algorithms to a matrix of pairwise distances estimated using a JTT model and then selecting the topology with the superior log likelihood value. The tree is drawn to scale, with the branch lengths measured in the number of substitutions per site. The analysis involved 10 amino acid sequences. All positions with less than 95% site coverage were eliminated. That is, fewer than 5% alignment gaps, missing data, and ambiguous bases were allowed at any position. There were a total of 372 positions in the final dataset. Evolutionary analyses were conducted in MEGA X [24].

Previous studies showed three microbial UGTs were validated with triterpenoid glycosylation activity, including BsYjiC (GenBank Protein Accession No. NP_389104) from *B. subtilis* 168 [19,20], UGT109A1 (GenBank Protein Accession No. ASY97769) from *B. subtilis* CTCG 63501 [21], and BsGT1 (GenBank Protein Accession No. ANP92054) from *B. subtilis* KCTC 1022 [22]. To classify which GT genes of *B. subtilis* ATCC 6633 are responsible for the biotransformation of GAA, a phylogenetic analysis of 30 annotated GTs from the *B. subtilis* ATCC 6633 genome (GenBank BioProject Accession No. PRJNA43011) was compared with the three validated microbial UGTs (Figure 2).

Among the 30 GTs, nine GT1 family genes (star in Figure 2), including two UGTs (*BsUGT398* and *BsUGT489*), were identified as putative gene candidates. The results revealed *BsUGT489* (GenBank Protein Accession No. WP_003220489) of *B. subtilis* ATCC 6633 was clustered with three validated UGTs (gray background in Figure 2). *BsUGT398* (GenBank Protein Accession No. WP_003225398), *BsGT110* (GenBank Protein Accession No. WP_003220110), *BsGT292* (GenBank Protein Accession No. WP_032727292), and *BsGT296* (GenBank Protein Accession No. WP_003219296) were closely related to the UGT cluster. Thus, the five candidates (bold text in Figure 2) were selected for further functional assay.

### 2.3. Cloning and Overexpression of GT1 from B. subtilis ATCC 6633 in E. coli

Among the five GT1 genes of *B. subtilis* ATCC 6633, three GT1 genes (*BsGT110*, *BsGT292*, *BsGT296*) had been previously subcloned into the pETDuet-1™ expression vector, overexpressed in *E. coli* BL-21 (DE3), and the produced GT1 proteins were purified with Ni^2+^ chelate affinity chromatography [25]. Thus, the three purified GT1 enzymes were ready for use in this study. The other two GT1 genes (*BsUGT398,* and *BsUGT489*) were newly amplified from *B. subtilis* ATCC 6633 genomic DNA with polymerase chain reaction (PCR) with specific primer sets (Table S1) and subcloned into the pETDuet-1 expression vector to form pETDuet-BsUGT (Figure S2a). The recombinant BsUGT was overexpressed in *E. coli*. The produced protein was purified and analyzed with sodium dodecyl sulfate polyacrylamide gel electrophoresis (SDS-PAGE) (Figure S2b). Thus, the five purified GT1 proteins were used for another biotransformation assay.

### 2.4. Activity Assays of GT1 from B. subtilis ATCC 6633 Toward GAA

**Figure 3:**
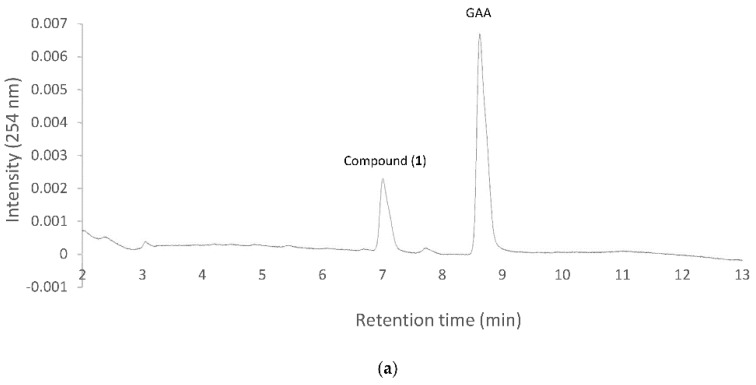

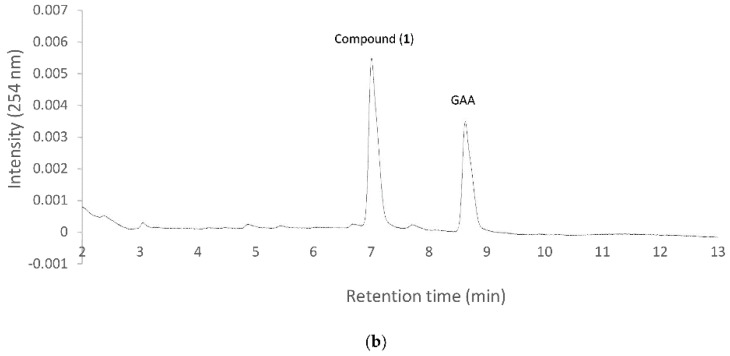
Biotransformation of GAA by purified BsUGT398 (**a**) or BsUGT489 (**b**). Two micrograms of purified BsUGT398 or BsUGT489 were incubated with 0.4 mM uridine diphosphate (UDP)-glucose and 0.02 mg/mL of GAA in the presence of 50 mM Tris pH 8.0 and 10 mM of MgCl_2_. After 30 min incubation, the mixtures were analyzed with UPLC. The UPLC conditions are described in Materials and Methods.

The five purified GT1 proteins were incubated with UDP-glucose and the precursor GAA. After a 30 min reaction, the reaction mixtures were analyzed with UPLC. Only BsUGT398 and BsUGT489 had biotransformation activity toward GAA, while the other three GT1 proteins of *B. subtilis* ATCC 6633 did not have this activity. Because our previous study showed that BsGT110 exhibited glycosylation activity toward 8-hydroxydaidzein [25], the reason for the lack of activity of the GT1 toward GAA is the substrate specificity. However, neither BsGT292 nor BsGT296 were active toward GAA and 8-hydroxydaidzein. The substrates of the two BsGTs are not known yet. The UPLC results further revealed the biotransformed metabolite in either the BsUGT398 (Figure 3a) or BsUGT489 (Figure 3b) reaction had an identical RT of 7.0 min, which was the same as the RT of compound (**1**) in the GAA biotransformation by the strain *B. subtilis* ATCC 6633 (Figure 1). Thus, this result implied that the BsUGT enzymes and the strain *B. subtilis* ATCC 6633 might have the same or similar biotransformation activity toward GAA.

In a previous study, we showed that *B. subtilis* ATCC 6633 could glycosylate antcin K, a major ergostane triterpenoid from the fruiting bodies of *Antrodia cinnamomea* [14]. Thus, we speculated that the five GT1 enzymes might have the ability to biotransform antcin K. The purified GT1 proteins were also incubated with UDP-glucose and the precursor antcin K. After a 30 min reaction, the reaction mixtures were analyzed with UPLC. Unfortunately, the results showed that no metabolite was biotransformed by the five GT1 proteins.

### 2.5. Optimal Catalyzing Conditions for BsUGT398 and BsUGT489

**Figure 4:**
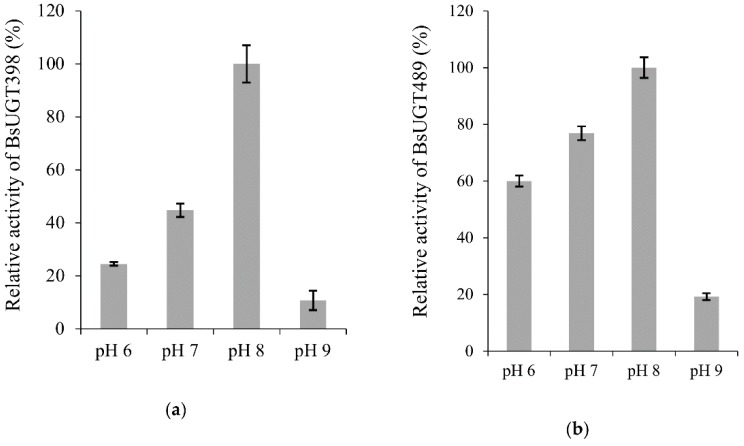

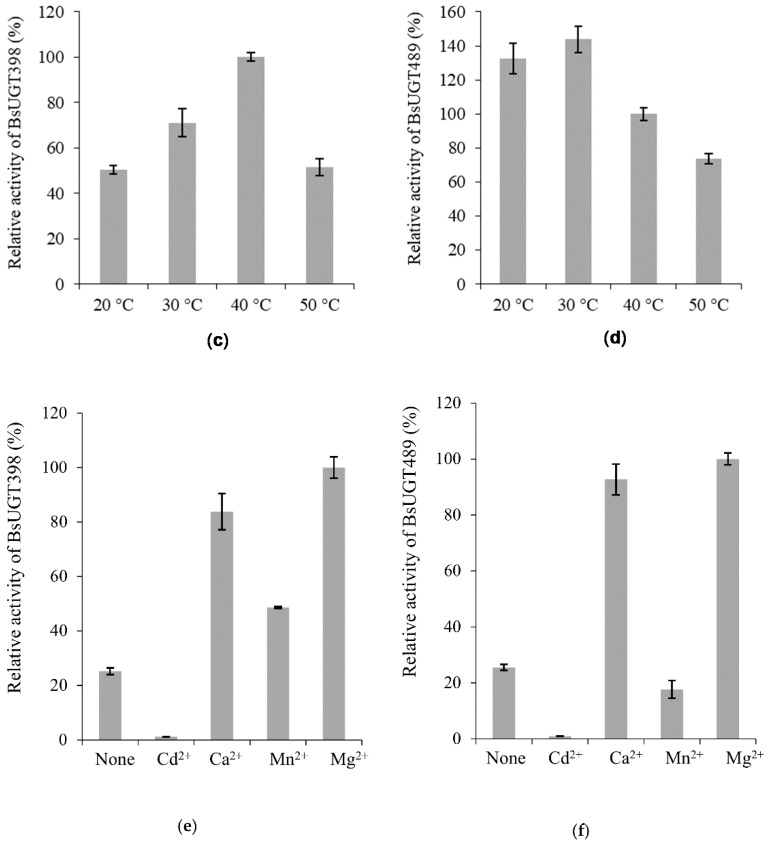
Effects of pH (**a**,**b**), temperature (**c**,**d**), and metal ion (**e**,**f**) on BsUGT398 (**a**,**c**,**e**) or BsUGT489 (**b**,**d**,**f**) activity. The standard condition was set as 2 µg of the purified enzyme, 1 mg/mL of GAA, 10 mM of MgCl_2_, and 10 mM of uridine diphosphate (UDP)-glucose in 50 mM of Tris at pH 8.0 and 40 °C. To determine the optimal reaction condition, the pH, temperature, or metal ion in the standard condition was replaced with the tested condition. Relative activity was obtained by dividing the area of the summation of the product peak of the reaction in the UPLC profile with that of the reaction at the standard condition. The mean (*n* = 3) is shown, and the standard deviations are represented by error bars.

The activities of purified BsUGT398 and BsUGT489 were determined at different pH values, temperatures, and metal ions. Many GTs utilize divalent metal ion cofactors, and Mg^2+^ was conjugated in the native crystals of some GTs [26]. In addition, Ca^2+^ and Mn^2+^ were also used in the crystal structure studies of GTs [27]. Therefore, we decided to use the three metal ions to optimize the activities of the two BsUGTs. On the other hand, to expand the testing range of the metal ions, a potentially inhibitory cation, Cd^2+^, was also used. The result is shown in Figure 4. The optimal catalyzing condition for each UGT protein is pH 8.0 with 10 mM of Mg^2+^ or Ca^2+^. The activity of the two UGTs decreased by about 70% in the absence of either the Mg^2+^ or Ca^2+^ metal ion and was completely inhibited in the presence of Cd^2+^. For temperature, BsUGT398 and BsUGT489 had optimal activity at 40 and 30 °C, respectively. 

### 2.6. Isolation and Identification of the Biotransformation Metabolite

**Figure 5:**
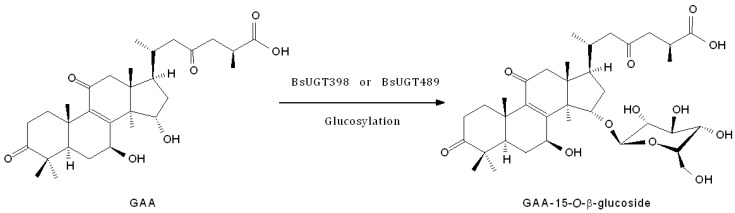
Biotransformation process of GAA by BsUGT398 or BsUGT489.

To resolve the chemical structures of the metabolites, the biotransformation was scaled up by either BsUGT398 or BsUGT489, and the metabolite from each biotransformation was purified with preparative high-performance liquid chromatography (HPLC). From a 40 mL reaction mixture containing 20 µg/mL of BsUGT, 1 mg/mL of GAA, 10 mM of UDP-glucose, 10 mM of MgCl_2_, and 50 mM of Tris pH 8.0, 15.1 mg and 37.4 mg of the metabolite, compound (**1**), were isolated from the biotransformation by BsUGT398 and BsUGT489, respectively. The metabolite from each biotransformation was elucidated with electrospray ionization mass spectrometry (ESI-MS) and nuclear magnetic resonance (NMR) spectra. They showed identical mass and ^13^C-NMR data. The mass data showed an [M − H]^−^ ion peak at *m*/*z*: 677.67 in the ESI-MS spectrum, corresponding to C_36_H_53_O_12_ that indicated the glucosylation of GAA. The structure of the metabolites was further confirmed as GAA-15-*O*-β-glucoside using NMR data, by comparison with that in our previously reported study [28]. The NMR spectrum of the two metabolites from either BsUGT398 or BsUGT489 are shown in Figure S3-S16.The biotransformation process of GAA by either BsUGT398 or BsUGT489, where d-glucose was tentatively assumed, is shown in Figure 5.

## 3. Discussion

Before the present study, three microbial UGTs—BsYjiC [19,20], UGT109A1 [21], and BsGT1 [22]—had been demonstrated to possess triterpenoid glycosylation activity. These UGTs catalyze the C-3, C-6, C-12, or C-20 carbon positions with a hydroxyl group for glycosylation of triterpenoids. Accordingly, BsUGT398 and BsUGT489 from *B. subtilis* ATCC 6633 that catalyzes the C-15 glycosylation of GAA are unique, and the glycosylation site on the triterpenoids is also new. Only one metabolite, glycosylation on the C-15 hydroxyl group, was biotransformed by the two BsUGTs, in spite of the other C-7 hydroxyl or C-26 carboxyl groups of GAA available for glycosylation (Figure 5). Moreover, neither of the two BsUGTs catalyzed the glycosylation of antcin K, which contains C-3, C-4, and C-7 hydroxyl and C-26 carboxyl groups but lacks the C-15 hydroxyl group available for glycosylation in the structure. The results imply that the two BsUGTs have regio- and substrate selection of glycosylation of triterpenoids. 

*B. subtilis* ATCC 6633 has been reported to possess glycosylation activity toward the pentacyclic triterpenoids oleanolic acid, echinocystic acid, or betulinic acid [29] or the C-26 carboxyl group of tetracyclic ergostane triterpenoid, antcin K [14]. Moreover, all the glycosylation activity by the strain were at carboxyl groups of the triterpenoid precursors. However, five GT1 enzymes of *B. subtilis* ATCC 6633 lacked biotransformation activity toward antcin K. Therefore, other enzymes must be responsible for catalyzing glycosylation on the carboxyl groups of the triterpenoids in the *B. subtilis* ATCC 6633. Nevertheless, previously identified triterpenoid-glycosylation UGT enzymes, including BsYjiC [19,20], UGT109A1 [21], BsGT1 [22], BsUGT398, and BsUGT489 (the present study), have been proven to catalyze the glycosylation of triterpenoids only on the hydroxyl groups of triterpenoids. Thus, the other GT family enzymes responsible for glycosylation on the carboxyl groups of triterpenoids remain unknown and should be investigated from the *B. subtilis* ATCC 6633 strain in the future. In addition to the GT families, some glycoside hydrolase (GH) families, such as the GH13 and GH70 families, have been reported to possess glycosyltransferase activity and accept small molecules, such as flavonoids, as sugar acceptors [17,30]. Thus, these GH enzymes of *B. subtilis* ATCC 6633 might also catalyze glycosylation on the carboxyl group of triterpenoids. Further studies to elucidate such catalytic enzymes will be undertaken in our laboratory.

Glycosylation is a common modification reaction in the biosynthesis of natural compounds. GT1 proteins are powerful enzymes in the glycosylation of natural compounds for in vivo or in vitro use. Glycosylation enhances the lipophilicity and water solubility of natural compounds. The development of a new biotransformation system of triterpenoid glycosylation would expand the search for new triterpenoids and the applications of the newly produced triterpenoids in medicine, cosmetics, and pharmacology use in the future.

## 4. Materials and Methods

### 4.1. Microorganism and Chemicals

*B. subtilis* ATCC 6633 (BCRC 10447) was purchased from BCRC (Food Industry Research and Development Institute, Hsinchu, Taiwan). Antcin K was obtained by the procedure in our previous study [14]. GAA was bought from Baoji Herbest Bio-Tech (Xi-An, Shaanxi, China). UDP-glucose was purchased from Cayman Chemical (Ann Arbor, MI, USA). The other chemicals, materials needed for PCR, restriction enzymes, reagents and solvents used were of high quality, and were purchased from commercially available sources.

### 4.2. Identification of Bacteria B. subtilis ATCC 6633 with Biotransformation Activity

*B. subtilis* ATCC 6633 was cultivated in 20 mL of a modified glucose-nutrient (MGN) medium which contained 5 g/L of peptone, yeast extract, K_2_HPO_4_, and NaCl; 20 g/L of glucose [14] and 20 mg/L of GAA. The cultivation was carried out at 180 rpm and 28 °C for 24 h. After cultivation, 1 mL of the culture was then mixed with an equal volume of methanol and analyzed with UPLC.

### 4.3. UPLC

The UPLC system (Acquity UPLC H-Class, Waters, Milford, MA, USA) was equipped with an analytic C18 reversed-phase column (Kinetex^®^ C18, 1.7 µm, 2.1 i.d. × 100 mm, Phenomenex Inc., Torrance, CA, USA). The operation conditions for UPLC of GAA and antcin K were from our previous study [14].

### 4.4. Expression and Purification of GT1 of B. subtilis ATCC 6633

The genomic DNA of *B. subtilis* ATCC 6633 was isolated using the commercial kit Geno *Plus*^TM^ (Viogene, Taipei, Taiwan). *BsUGT398* and *BsUGT489* of *B. subtilis* ATCC 6633 were amplified from the genomic DNA with PCR with specific primer sets (Table S1). The amplified GT1 genes were subcloned into the pETDuet-1™ vector through suitable restriction enzyme sites (Table S1) to obtain the expression vector pETDuet-UGT (Figure S2a). The expression vectors were transformed into *E. coli* BL21 (DE3) via electroporation to obtain recombinant *E. coli*. 

Recombinant BsUGT398 and BsUGT489 were produced and purified from the recombinant *E. coli.* The experimental procedure was the same as our previous study [25]. 

### 4.5. In Vitro Biotransformation Assay

In vitro biotransformation was performed by purified GT1 protein. In 1 mL reaction mixture, 2 µg of purified GT1 protein, 0.02 mg/mL of GAA or antcin K, 0.4 mM of UDP-glucose, 10 mM of MgCl_2_, and 50 mM of Tris pH 8.0 were added. The reaction was carried out at 40 °C for 30 min. After the reaction, the mixture was stopped by adding an equal volume of methanol and analyzed with UPLC. For the optimal experiments, 2 µg of the purified BsUGT398 or BsUGT489, 1 mg/mL of GAA, and 10 mM of UDP-glucose were used. The pH, temperature, or metal ion described above was replaced by the tested condition. For pH testing, phosphate buffer at pH 6.0 and 7.0) and Tris buffer at pH 8.0 and 9.0 were used. For metal ion testing, 10 mM of MgCl_2_, CaCl_2_, CdSO_4_ or MnCl_2_ was used. The relative activity was obtained by dividing the area of the product peak of the reaction in the UPLC profile by that of the reaction at Tris pH 8.0 and 10 mM of MgCl_2_. 

### 4.6. Scale-Up, Isolation, and Identification of the Biotransformation Product

To purify the biotransformation metabolite, the reaction was scaled up to a 40 mL reaction mixture containing 20 µg of purified BsUGT398 or BsUGT489, 1 mg/mL of GAA, 10 mM of UDP–glucose, 10 mM of MgCl_2_, and 50 mM of Tris pH 8.0. After reaction at 30 °C (BsUGT489) or 40 °C (BsUGT398) for 60 min, 40 mL of methanol was added to stop the reaction. The reaction mixture was filtrated through a 0.2 µm nylon membrane, and applied into a preparative YoungLin HPLC system (YL9100, YL Instrument, Gyeonggi-do, South Korea). The system was equipped with a preparative C18 reversed-phase column (Inertsil, 10 µm, 20.0 i.d. × 250 mm, ODS 3, GL Sciences, Eindhoven, The Netherlands). The operational conditions for the preparative HPLC were the same as those in UPLC. The elution corresponding to the peak of the metabolite in UPLC was collected, concentrated under vacuum, and then lyophilized. Finally, 15.1 mg and 37.6 mg of compound (**1**) were obtained from the BsUGT398 and BsUGT489 biotransformation, respectively, and the structure of the compound was confirmed with NMR and mass spectrometry analysis. The mass spectrometry analysis was performed on a Finnigan LCQ Duo mass spectrometer (ThermoQuest Corp., San Jose, CA, USA) with electrospray ionization (ESI). For the NMR experiments, purified samples quantities ranged from 4 mg to 8 mg and were dissolved in 400 µL pyridine-*d*_5_, and ^1^H- and ^13^C-NMR, and distortionless enhancement by polarization transfer (DEPT), heteronuclear single quantum coherence (HSQC), heteronuclear multiple bond connectivity (HMBC), correlation spectroscopy (COSY), and nuclear Overhauser effect spectroscopy (NOESY) spectra were recorded on a Bruker AV-700 NMR spectrometer (Bruker Corp., Billerica, MA, USA) at ambient temperature. Standard pulse sequences and parameters from the Bruker library were used for the NMR experiments, and all chemical shifts were reported in parts per million (ppm, δ).

### 4.7. Phylogenetic Analysis of GTs from B. subtilis ATCC 6633

The unrooted phylogenetic tree of these candidate GT protein sequences was constructed with the maximum likelihood method, using Molecular Evolutionary Genetics Analysis (MEGA X) software [24] with 500 bootstrap replications, the general reversible mitochondrial model [23], and partial deletion. 

## 5. Conclusions

Two new BsUGTs from *B. subtilis* ATCC 6633 were identified as transferring a glucose moiety to GAA, producing a new triterpenoid glucoside, GAA-15-*O*-β-glucoside. More than 10,000 microbial GT genes have been deposited in the CAZy database. However, less than 1% of these GT genes have been functionally characterized. The two BsUGTs were newly identified with novel triterpenoid glycosylation activity. The two *B. subtilis* BsUGTs are the only identified GTs with glycosylation activity toward *Ganoderma* triterpenoids. Thus, the present study has developed a novel biotransformation system which might be applied to numerous *Ganoderma* triterpenoids to create many new *Ganoderma* triterpenoid glucosides in the future.

## Supplementary Materials

Supplementary materials can be found at http://www.mdpi.com/1422-0067/19/11/3469/s1. Table S1: Nucleotide sequences of the primers used for amplification of GT1 in the present study. Figure S1: UPLC of the 24 h fermentation broth using the *B. subtilis* ATCC 6633 strain without the addition of GAA. Figure S2: Expression of BsUGT398 and BsUGT489 from *B. subtilis* ATCC 6633 in *E. coli*. Figure S3. The ^1^H-NMR (700 MHz, pyridine-*d*_5_) spectrum of metabolite derived from GAA biotransformation by BsUGT398. Figure S4. The ^13^C-NMR (176 MHz, pyridine-*d*_5_) spectrum of metabolite derived from GAA biotransformation by BsUGT398. Figure S5. The DEPT-90 and DEPT-135 (176 MHz, pyridine-*d*5) spectrum of metabolite derived from GAA biotransformation by BsUGT398. Figure S6. The HSQC (700 MHz, pyridine-*d*_5_) spectrum of metabolite derived from GAA biotransformation by BsUGT398. Figure S7. The HMBC (700 MHz, pyridine-*d*_5_) spectrum of metabolite derived from GAA biotransformation by BsUGT398. Figure S8. The ^1^H-^1^H COSY (700 MHz, pyridine-*d*_5_) spectrum of metabolite derived from GAA biotransformation by BsUGT398. Figure S9. The NOESY (700 MHz, pyridine-*d*_5_) spectrum of metabolite derived from GAA biotransformation by BsUGT398. Figure S10. The ^1^H-NMR (700 MHz, pyridine-*d*_5_) spectrum of metabolite derived from GAA biotransformation by BsUGT489. Figure S11. The ^13^C-NMR (176 MHz, pyridine-*d*_5_) spectrum of metabolite derived from GAA biotransformation by BsUGT489. Figure S12. The DEPT-90 and DEPT-135 (176 MHz, pyridine-*d*5) spectrum of metabolite derived from GAA biotransformation by BsUGT489. Figure S13. The HSQC (700 MHz, pyridine-*d*_5_) spectrum of metabolite derived from GAA biotransformation by BsUGT489. Figure S14. The HMBC (700 MHz, pyridine-*d*_5_) spectrum of metabolite derived from GAA biotransformation by BsUGT489. Figure S15. The ^1^H-^1^H COSY (700 MHz, pyridine-*d*_5_) spectrum of metabolite derived from GAA biotransformation by BsUGT489. Figure S16. The NOESY (700 MHz, pyridine-*d*_5_) spectrum of metabolite derived from GAA biotransformation by BsUGT489.

## Abbreviations

GAA	Ganoderic acid A
GT	Glycosyltransferase
GT1	Glycosyltransferase family 1
UDP	Uridine diphosphate
UGT	Uridine diphosphate-dependent glycosyltransferases
