# Supplementary material for: Uridine Diphosphate-Dependent Glycosyltransferases from Bacillus subtilis ATCC 6633 Catalyze the 15-O-Glycosylation of Ganoderic Acid A
Source: Int J Mol Sci. 2018 Nov 5;19(11):3469. doi: 10.3390/ijms19113469 (PMC6275011; doi:10.3390/ijms19113469)

Uridine Diphosphate-Dependent Glycosyltransferases from *Bacillus subtilis* ATCC 6633 Catalyzing 15-*O*-Glycosylation of Ganoderic Acid A

Te-Sheng Chang ^1,+^, Jiumn-Yih Wu ^2,+^, Tzi-Yuan Wang ^3^, Kun-Yuan Wu ^1^, and Chien-Min Chiang ^4,*^

**Table S1.** Nucleotide sequences of the primers used for amplifications of GT1 in the present study.

| **Name** | **Nucleotide sequence of the primer ^1^** |
| --- | --- |
| BsUGT398F-EcoRI | CGCGAATTCgaagacagtattgattttaaattttcc |
| BsUGT398R-XhoI | CGCCTCGAGttattttctctgttctataaattgatc |
| BsUGT489F-EcoRI | CGCGAATTCGatgaaaaagcaccatatttcgatg |
| BsUGT489R-XhoI | CGCCTCGAGttattgcggtacagcggatttttt |

^1^ The sequences for the designed restriction enzyme sites are noted by underline.

**Figure S1.** UPLC analysis of the 24 h fermentation broth using *B. subtilis* ATCC 6633 strain without the addition of GAA.

| 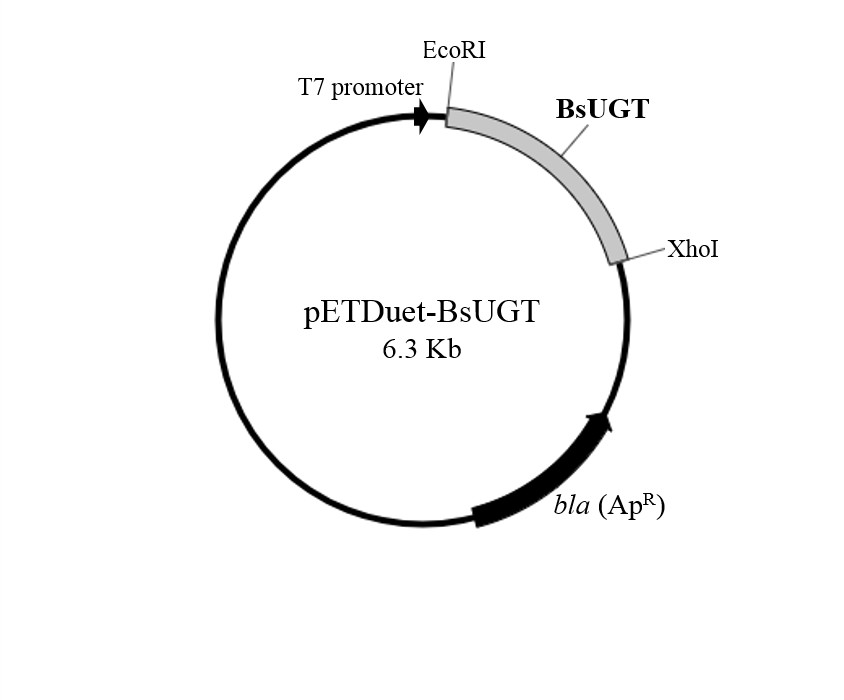 |
| --- |
| (**a**) |
| 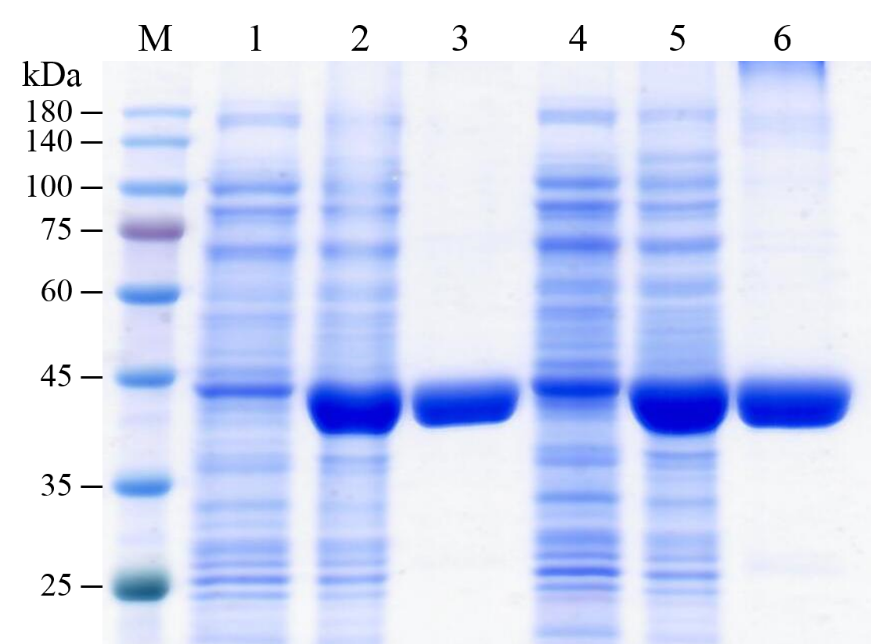 |
| (**b**) |

**Figure S2.** Expression of BsUGT398 and BsUGT489 from *B. subtilis* ATCC 6633 in *E. coli*. (**a**) Diagram of the recombinant repression plasmid. (**b**) SDS-PAGE analysis of expressed and purified proteins from recombinant *E. coli* harboring pETDuet-*BsUGT398* (lane 1 to lane 3) or pETDuet-*BsUGT489* (lane 4 to lane 6). Lane M: molecular marker; lane 1 and lane 4: total protein before induction; lane 2 and lane 5: total protein after 20 h induction; lane 3 and lane 6: purified protein.


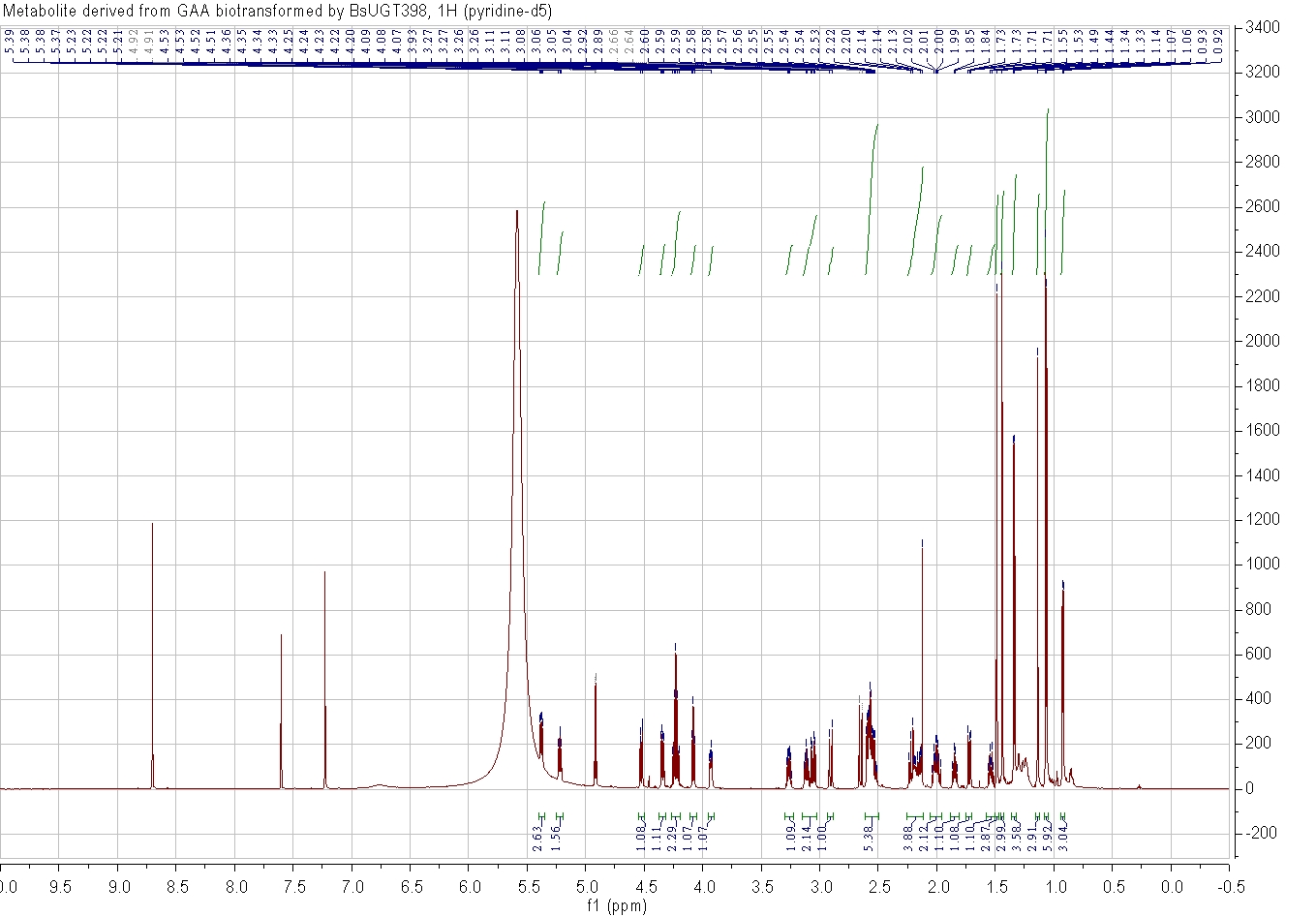


**Figure S3.** The ^1^H-NMR (700 MHz, pyridine-*d*_5_) spectrum of metabolite derived from GAA biotransformation by BsUGT398..


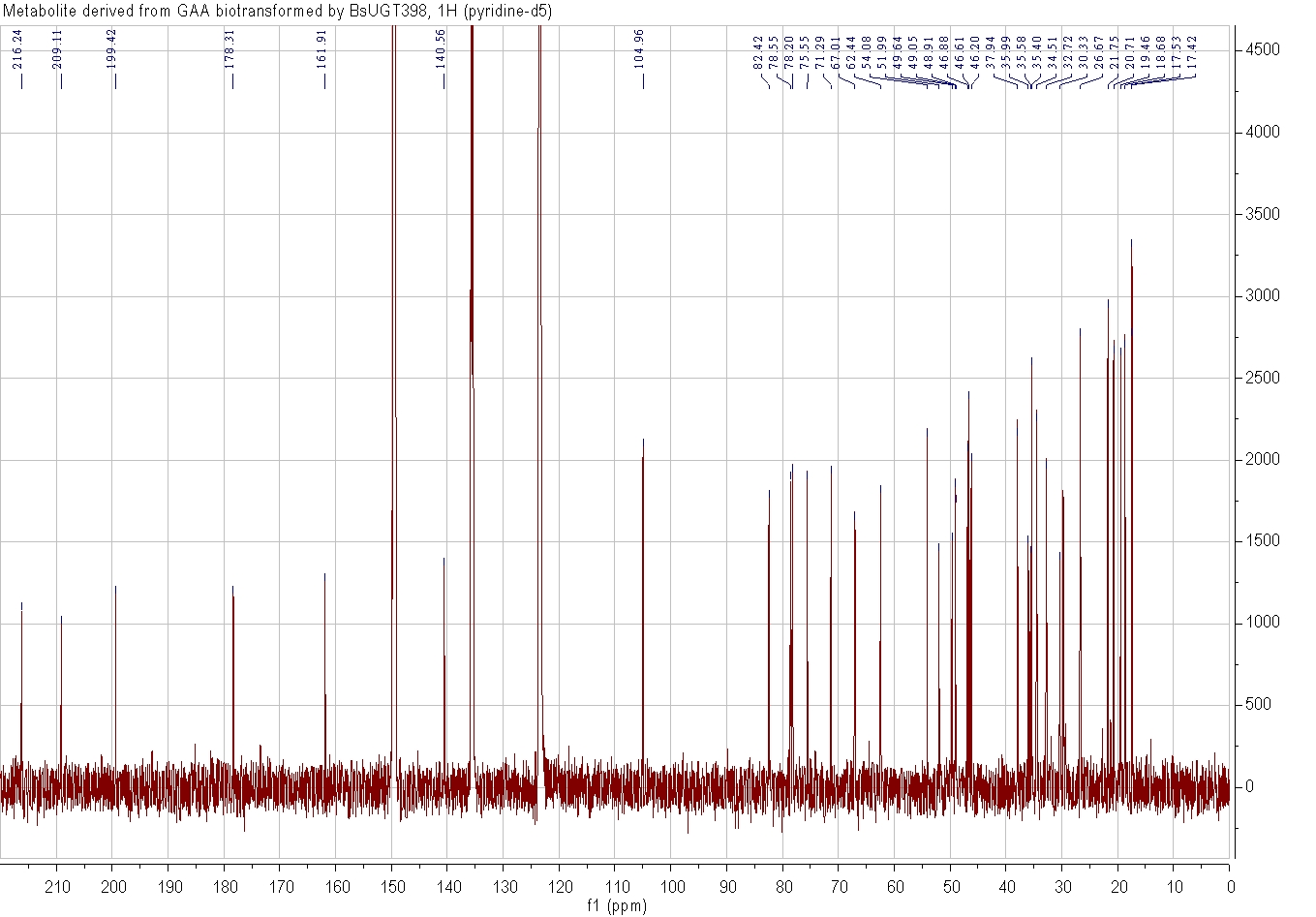


**Figure S4.** The ^13^C-NMR (176 MHz, pyridine-*d*_5_) spectrum of metabolite derived from GAA biotransformation by BsUGT398..


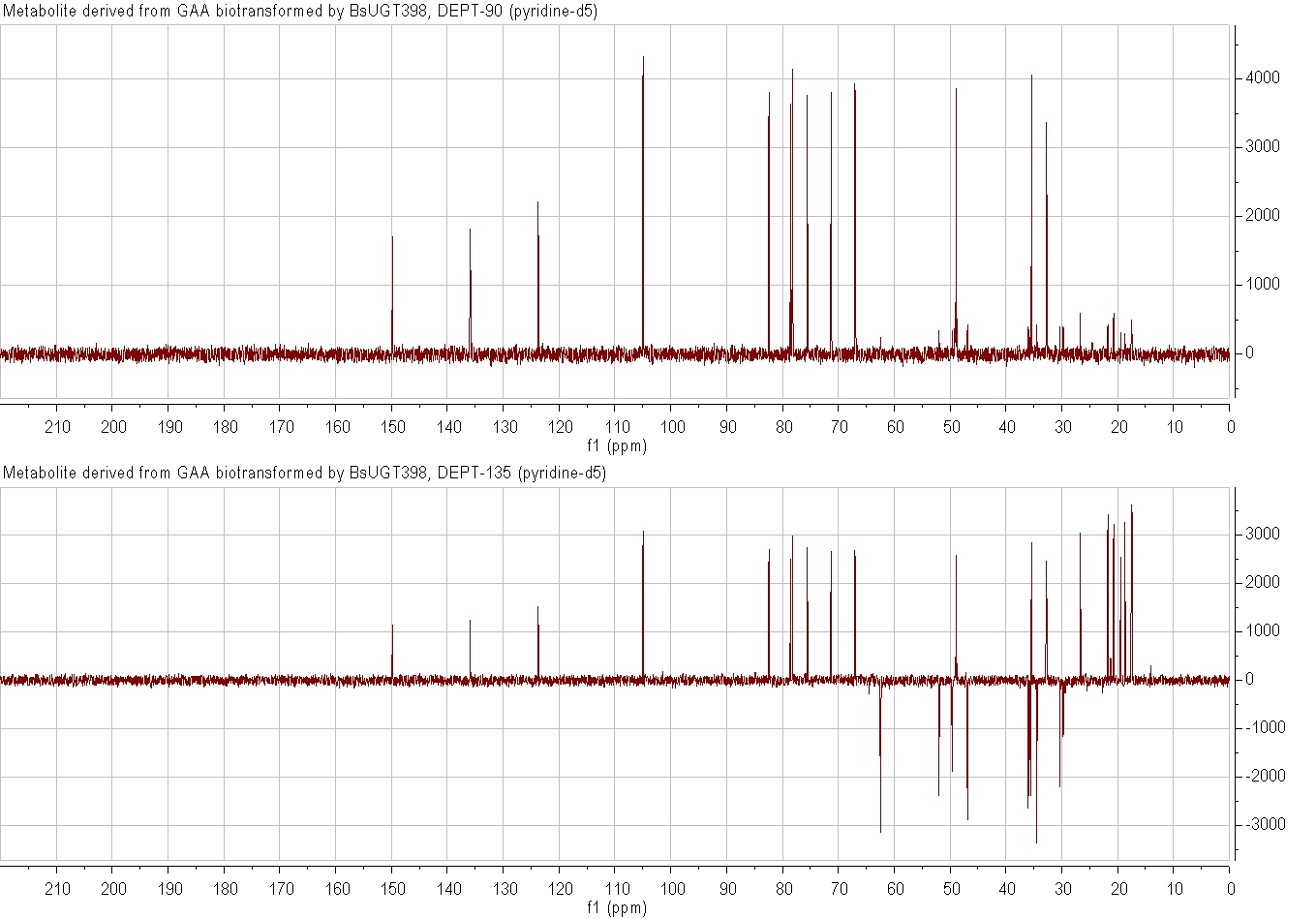


**Figure S5.** The DEPT-90 and DEPT-135 (176 MHz, pyridine-*d*5) spectrum of metabolite derived from GAA biotransformation by BsUGT398..


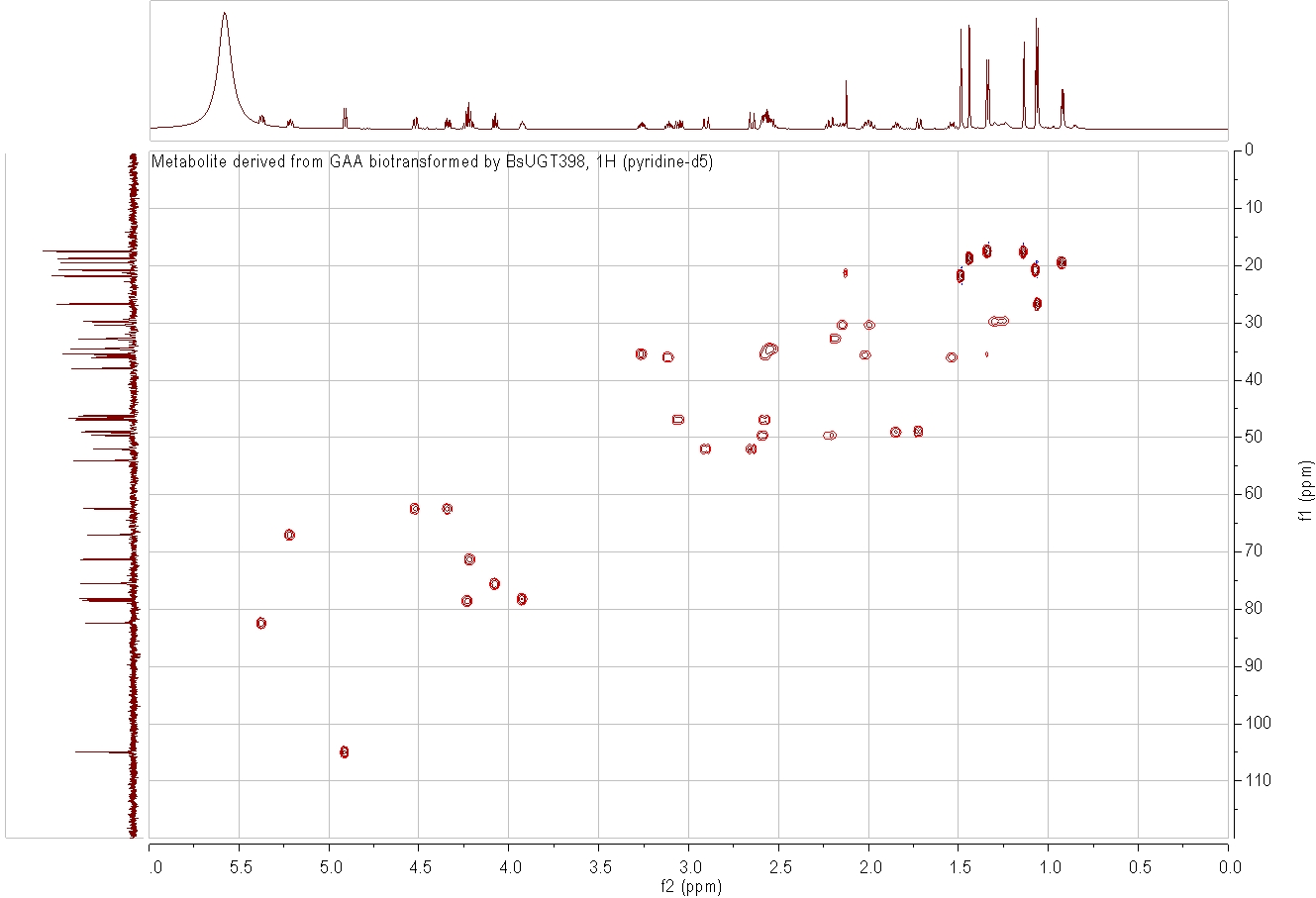


**Figure S6.** The HSQC (700 MHz, pyridine-*d*_5_) spectrum of metabolite derived from GAA biotransformation by BsUGT398..


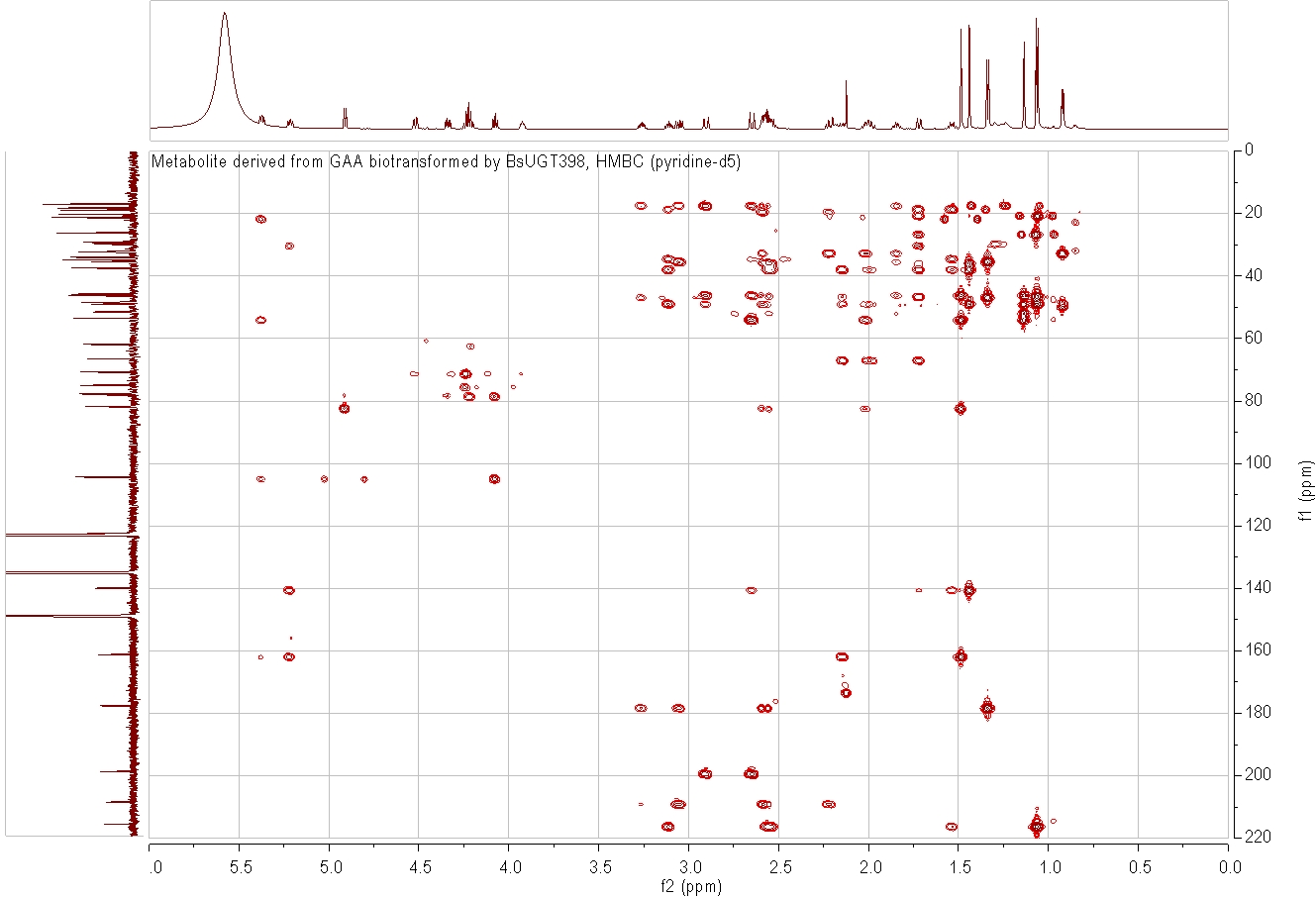


**Figure S7.** The HMBC (700 MHz, pyridine-*d*_5_) spectrum of metabolite derived from GAA biotransformation by BsUGT398..


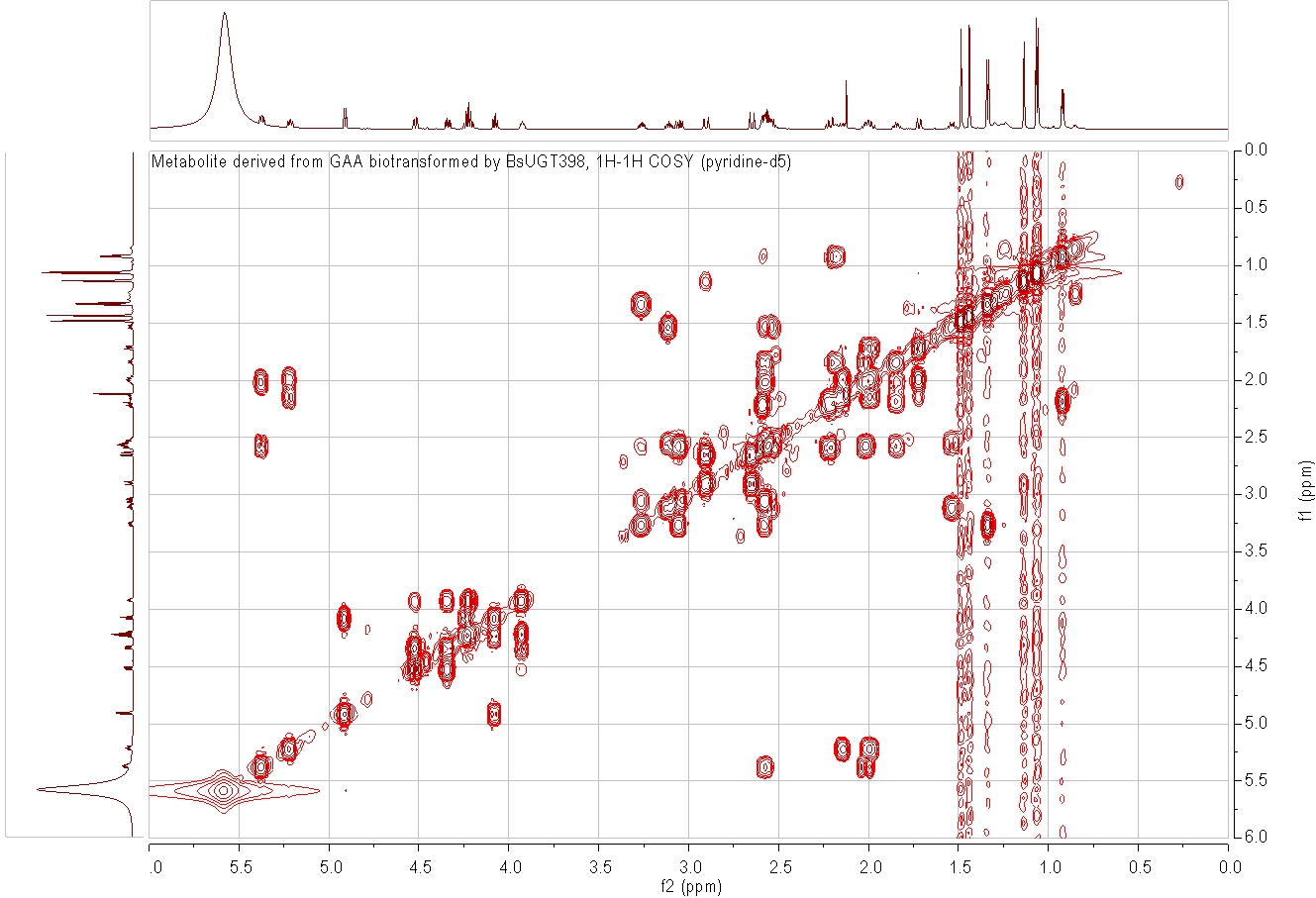


**Figure S8.** The ^1^H-^1^H COSY (700 MHz, pyridine-*d*_5_) spectrum of metabolite derived from GAA biotransformation by BsUGT398..


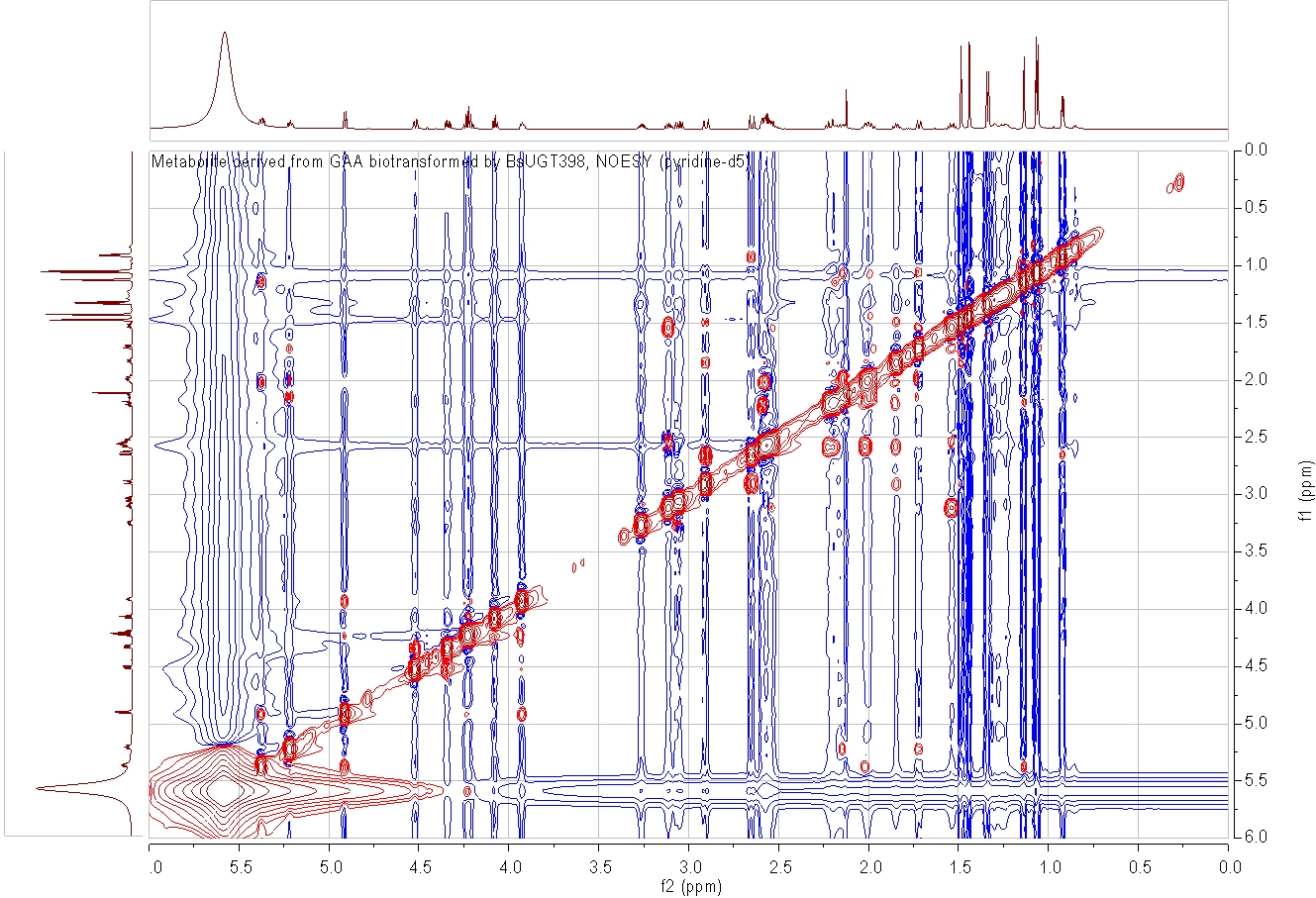


**Figure S9.** The NOESY (700 MHz, pyridine-*d*_5_) spectrum of metabolite derived from GAA biotransformation by BsUGT398. .


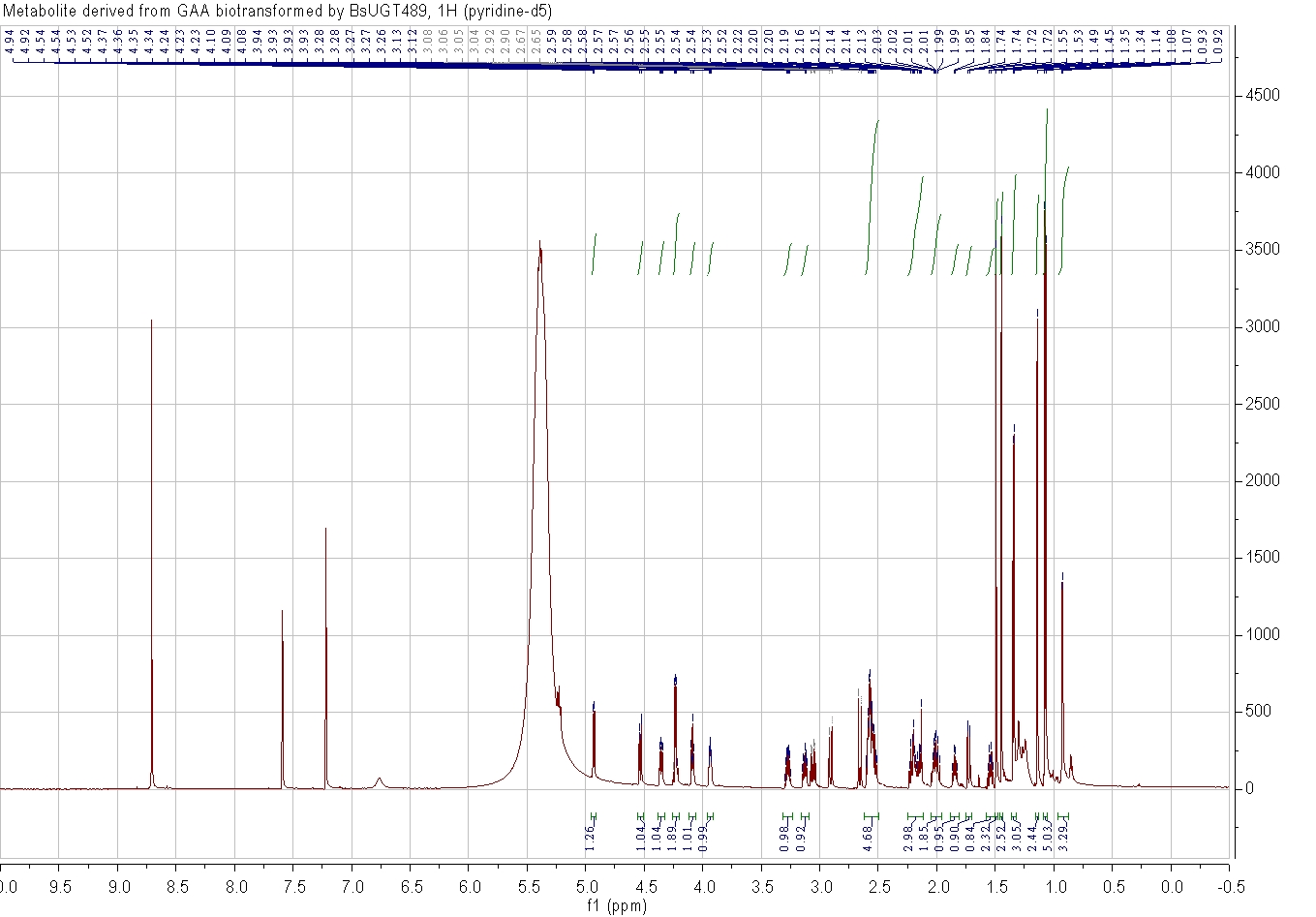


**Figure S10.** The ^1^H-NMR (700 MHz, pyridine-*d*_5_) spectrum of metabolite derived from GAA biotransformation by BsUGT489..


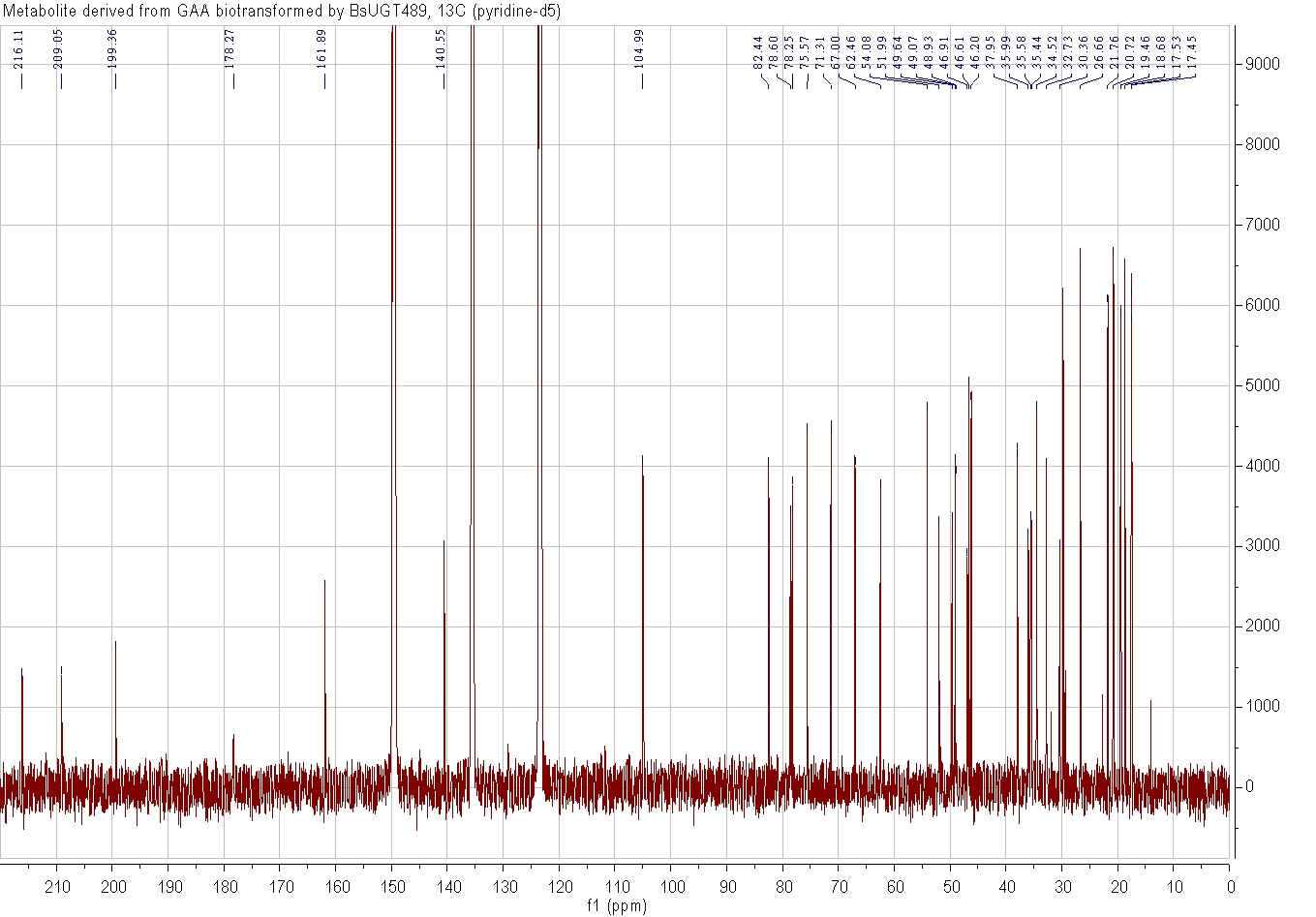


**Figure S11.** The ^13^C-NMR (176 MHz, pyridine-*d*_5_) spectrum of metabolite derived from GAA biotransformation by BsUGT489..


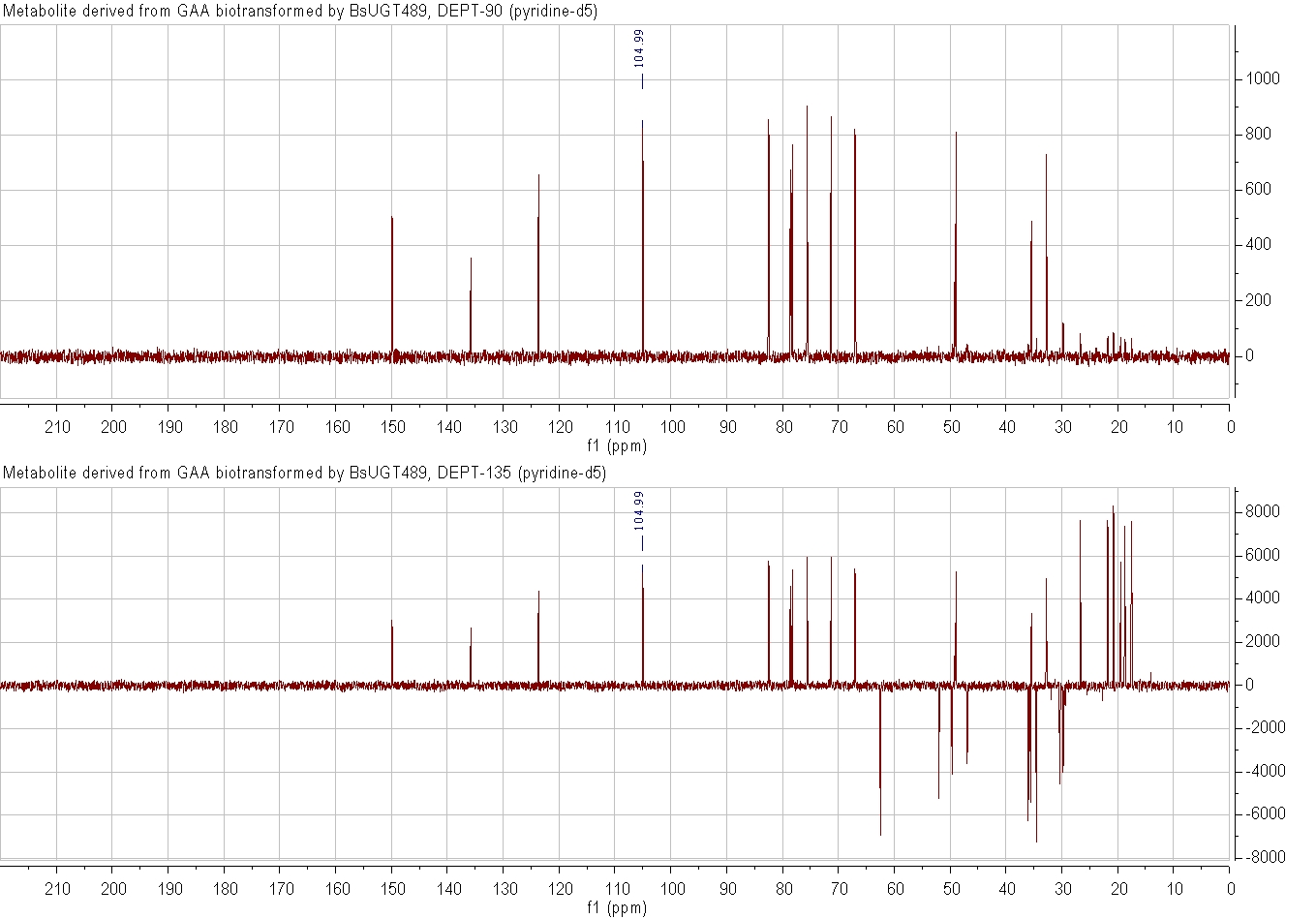


**Figure S12.** The DEPT-90 and DEPT-135 (176 MHz, pyridine-*d*5) spectrum of metabolite derived from GAA biotransformation by BsUGT489..


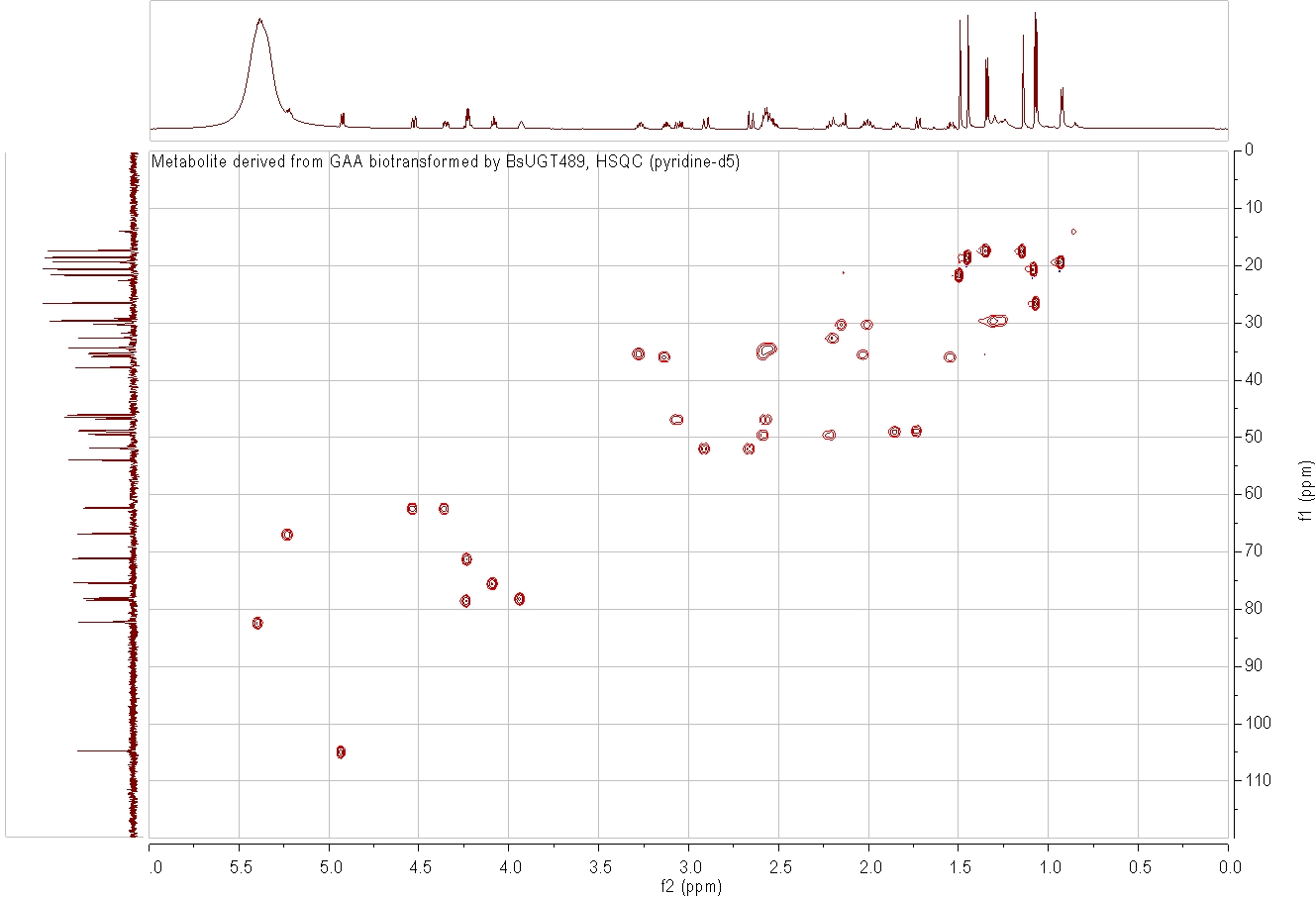


**Figure S13.** The HSQC (700 MHz, pyridine-*d*_5_) spectrum of metabolite derived from GAA biotransformation by BsUGT489..


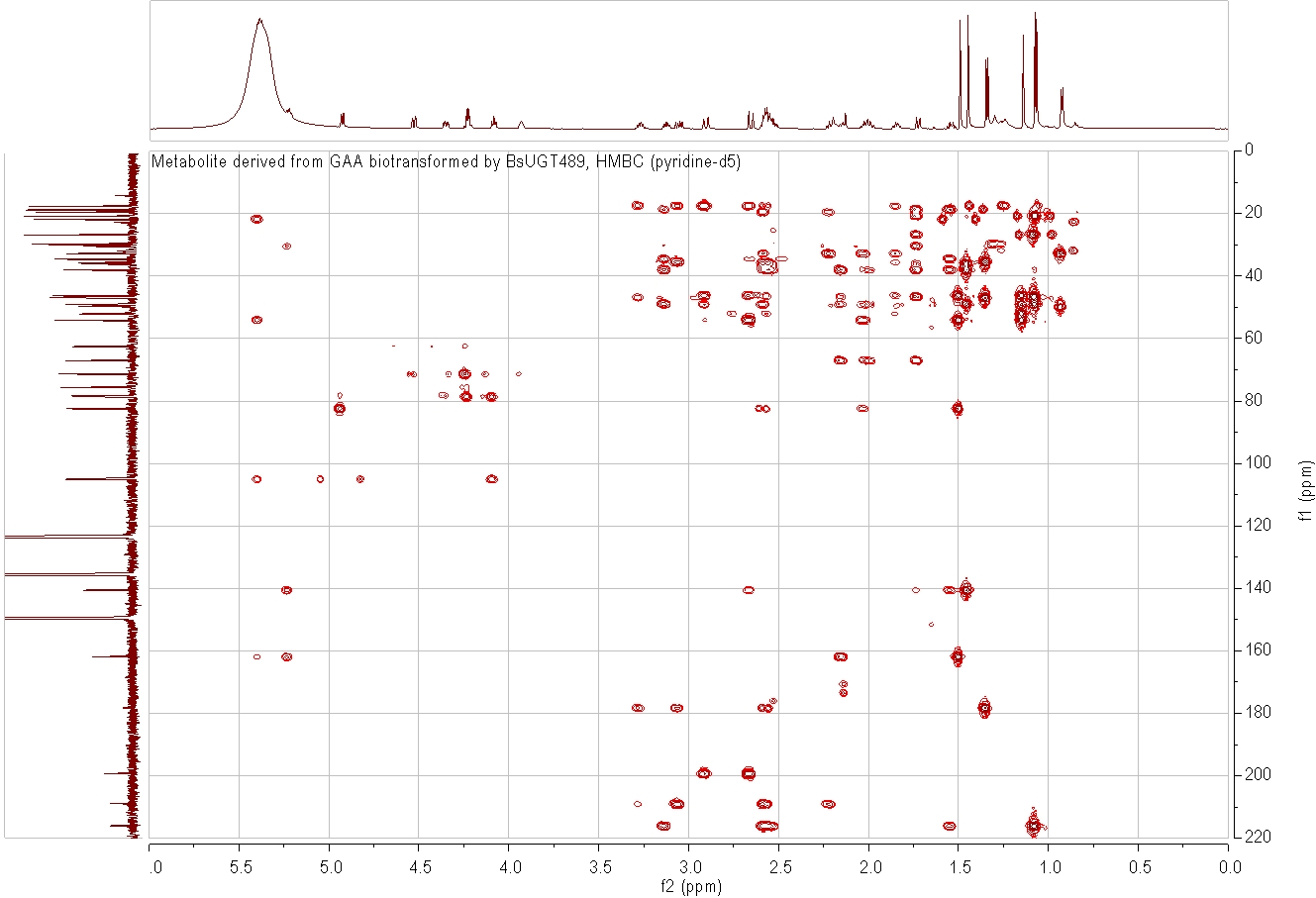


**Figure S14.** The HMBC (700 MHz, pyridine-*d*_5_) spectrum of metabolite derived from GAA biotransformation by BsUGT489..


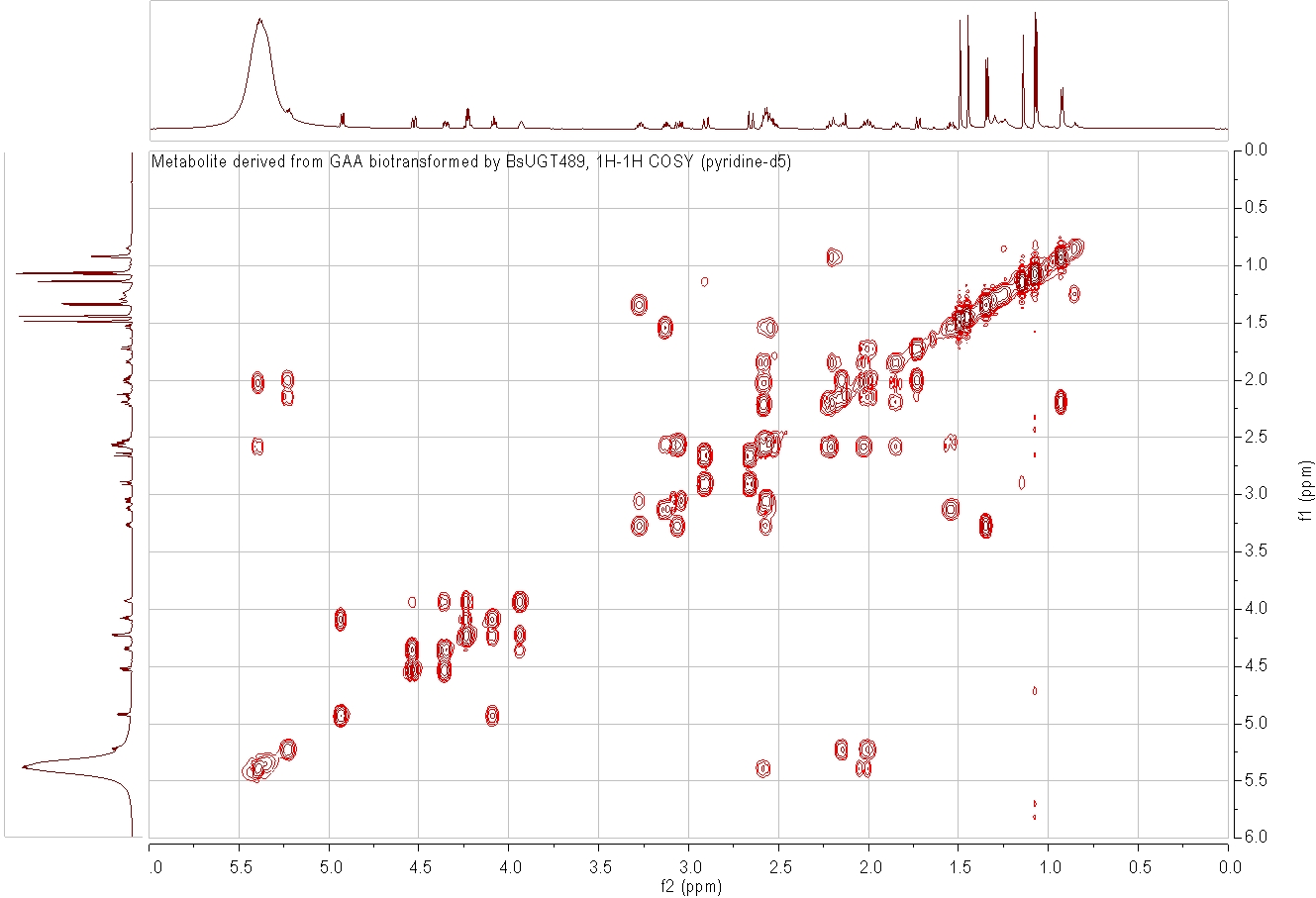


**Figure S15.** The ^1^H-^1^H COSY (700 MHz, pyridine-*d*_5_) spectrum of metabolite derived from GAA biotransformation by BsUGT489..


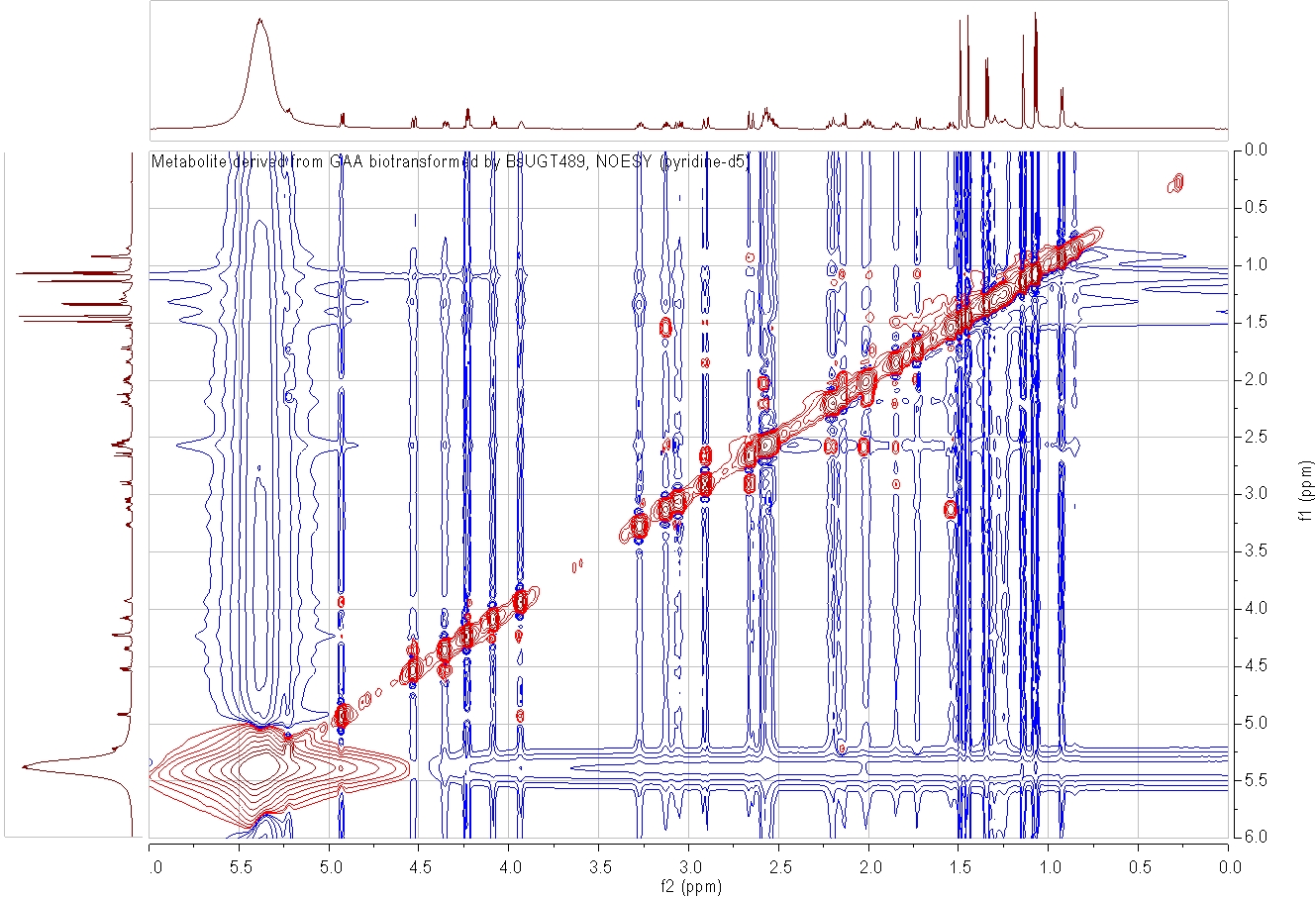


**Figure S16.** The NOESY (700 MHz, pyridine-*d*_5_) spectrum of metabolite derived from GAA biotransformation by BsUGT489.

© 2018 by the authors. Licensee MDPI, Basel, Switzerland. This article is an open access article distributed under the terms and conditions of the Creative Commons Attribution (CC BY) license (http://creativecommons.org/licenses/by/4.0/).
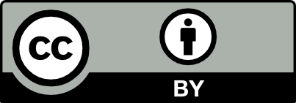

Supplement: Supplementary file 1 [file ijms-19-03469-s001.zip › ijms-378521 suppl final check.docx]
